# Clinical characteristics and prognosis of pulmonary large cell carcinoma: A population‐based retrospective study using SEER data

**DOI:** 10.1111/1759-7714.13420

**Published:** 2020-04-16

**Authors:** Liu Xiaochuan, Yu Jiangyong, Zhang Ping, Wu Xiaonan, Li Lin

**Affiliations:** ^1^ Department of Medical Oncology Beijing Hospital, National Center of Gerontology; Institute of Geriatric Medicine, Chinese Academy of Medical Sciences Beijing China; ^2^ Peking University Fifth School of Clinical Medicine Beijing China

**Keywords:** Clinical characteristics, prognosis, pulmonary large cell carcinoma, seer, treatment

## Abstract

**Background:**

Pulmonary large cell carcinoma (LCC) is an infrequent neoplasm with a poor prognosis. This study explored the clinical characteristics and survival prognostic factors of LCC patients.

**Methods:**

Patient data were extracted from the Surveillance, Epidemiology, and End Results (SEER) database. Chi‐square tests or rank‐sum tests were used to compare differences in clinical characteristics. Log‐rank tests, univariate, and multivariate analyses were performed to investigate the independent factors of survival. Analyses of stage I–IV patients were performed to further explore the optimal treatment.

**Results:**

In total, 3197 LCC patients were included in this analysis. Compared with other non‐small cell lung cancers (NSCLCs), there was a worse overall survival (OS) from LCC. LCC was more common in males, over age 60 and in the upper lobe. A total of 73.6% of patients were stage III/IV. The median OS of stage I–IV patients was 42 months, 22 months, 11 months, and three months, respectively. The elderly, males, later stage, and main bronchus location, or overlapping lesions were risk factors for survival prognosis. In stage I–III patients, treatment including surgery could significantly reduce the risk of death by 60% at least compared with no therapy. Surgery was still beneficial for stage IV patients, and the hazard ratio (HR) compared with no therapy was 0.462 (*P* = 0.001).

**Conclusions:**

Our study concluded that LCC has unique clinical features, and that age, sex, primary site, stage, and treatment are significantly related to OS. Surgery based comprehensive treatments are effective for LCC.

**Key points:**

**Significant findings of the study** In stage IV patients, chemotherapy or radiotherapy combined with surgery could further improve survival. When surgical resection involved more than one lobe, it may be beneficial for survival prognosis.

**What this study adds** LCC patients were principally male and over age 60, with later stages and poor survival prognosis. Age, sex, stage, primary site and therapy are closely related to survival.

## Introduction

The morbidity and mortality of patients with lung cancer are the highest in cancer worldwide,^1^ in which non‐small cell lung cancer (NSCLC) accounts for 85%.^2^ According to the WHO 2004 classification criteria, LCC, taking possession of 1.4% of all cases,^3^ was defined as NSCLC that lacked adenoidal or squamous morphology features, including several subtypes, such as large cell neuroendocrine carcinoma (LCNEC), lymphoepithelioma‐like carcinoma (LELC), basaloid carcinoma, clear cell carcinoma, and large cell carcinoma with rhabdoid phenotype.^4^ Gradually, researchers have found that LCC could be reclassified as adenocarcinoma (LCC‐ADE), squamous cell carcinoma (LCC‐SCC), or marker‐null (LCC‐null) based on different immunohistochemical (IHC) characteristics. In addition, disease‐free survival and OS of LCC‐null were significantly lower than LCC‐ADC and LCC‐SCC.^5^ Therefore, the classification standard of WHO in 2015 has obviously changed. Depending on the outcome of TTF‐1 or P40 staining, solid‐grown tumors are classified as solid adenocarcinoma or nonkeratinizing squamous cell carcinoma. Other subtypes of LCC have also been reclassified into other pathological types.^6^

To date, there is still little information about the clinical characteristics and prognosis of patients with LCC. Therefore, we conducted a retrospective analysis of LCC in the SEER database, which collects cancer information from various registries covering nearly 34.6% of the U.S. population.^7^ In this study, we explored the clinical features and factors related to patients’ survival from LCC, and constructed a nomogram to predict three‐ and five‐year overall survival.

## Methods

### Ethical statement

We signed the SEER Research Data Agreement and obtained permission to access and use the data from the public SEER database. Our research was therefore exempt by the local ethics committee.

### Data extraction

Patient data were extracted from Incidence‐SEER 18 Regs Research Data+Hurricane Katrina Impacted Louisiana Cases, Nov 2018 Sub (1975–2016 varying), using SEER*Stat software version 8.3.6. Inclusion criteria were: (i) “Lung and Bronchus” restricted by site recode ICD‐O‐3/WHO2008; (ii) 8012 (large cell carcinoma), 8013 (large cell neuroendocrine carcinoma), 8070 (squamous cell carcinoma), 8083 (basaloid squamous cell carcinoma) and 8140 (adenocarcinoma) by histologic type ICD‐O‐3; (iii) “One primary only” by sequence number; and (iv) 2004–2015 by year at diagnosis, in which there was enough information to stage according to latest standard. The following covariates were collected from the database: age at diagnosis, sex, race, pathological type, primary site, grade, information about American Joint Committee on Cancer (AJCC) TNM stage, therapy, and survival data. Following the guidelines of the AJCC Cancer Staging Manual eighth edition,^8^ T stage was evaluated by tumor size, tumor extension, CS site‐specific factor‐1, and CS site‐specific factor‐2. N stage was evaluated by CS lymph nodes. M stage was evaluated by CS Mets at dx. Samples were excluded without complete information. Finally, 3197 patients with LCC, 1111 with LCNEC, 189 with BSC, 40216 with SCC, and 59 772 with ADE were included.

### Statistical analysis

Groups were compared by Chi‐square tests or Fisher's exact tests for nominal variables and by Wilcoxon tests or Kruskal‐Wallis tests for ordered categorical variables. Kaplan‐Meier survival curves were constructed and compared using log‐rank tests for LCC patients. The Bonferroni method was used to correct multiple comparisons, and the level of significance was set at 0.05. Then prognostic factors were identified by Cox proportional hazards regression and reported as hazard ratios (HRs). Factors with *P*‐value <0.05 in univariate analysis (UVA) were included in multivariate analysis (MVA). The nomogram was derived from the results of MVA. The Bootstrap method was used for internal verification of the nomogram and times of repeat samples were 1000. The concordance index (C‐index) was used to evaluate the discriminative ability and calibration plots were used to test for consistency. The range of the C‐index was 0–1. The closer C‐index was to 1, the better the model was for discrimination. If the slope of the calibration plot was close to 1, it would show a high consistency between predicted and actual survival proportions. Univariate analyses were performed to explore the optimal treatment in stage I–IV patients and reported as hazard ratios (HRs). Statistical analyses were performed using R 3.6.1 and SPSS 24.

## Results

### Comparison of patient characteristics between LCC and other NSCLCs

As shown in Table 1, a total of 3197 LCC patients were involved in the analysis, of which 2185 cases met the TNM clinical staging criteria, and 1012 cases met the pathological staging criteria. Approximately 40% of patients were over 70‐years‐old at diagnosis. The number of male patients was 1891 (59.1%), more than female patients. The vast majority of patients were poorly differentiated or undifferentiated, accounting for 98.4%. White people were the most of all races. The most common LCC site was the upper lobe in 1861 patients (58.2%), followed by the lower lobe in 761 patients (23.8%). Most were N positive (58.4%) and stage III/IV (73.6%). Data on metastasis were available since 2010. There were 385 stage IV patients with complete metastasis information. The rate of bone metastasis, 36.4%, was the highest, followed by 30.1% of the brain, 27.5% of the lung, and 19.0% of the liver.

**Table 1:** Comparison between LCC and other NSCLCs

Variables	LCC	LCNEC	BSC	SCC	ADE
Total, n (%)	3197 (100)	1111 (100)	189 (100)	40 216 (100)	59 772 (100)
Age, n (%)					
≤70	1931 (60.4)	748 (67.3)*	91 (48.1)*	21 141 (52.6)*	36 400 (60.9)
>70	1266 (39.6)	363 (32.7)	98 (51.9)	19 075 (47.4)	23 372 (39.1)
Sex, n (%)					
Female	1306 (40.9)	504 (45.4)*	77 (40.7)	14 904 (37.1)*	31 103 (52.0)*
Male	1891 (59.1)	607 (54.6)	112 (59.3)	25 312 (62.9)	28 669 (48.0)
Race, n (%)					
White	2571 (80.4)	941 (84.7)*	159 (84.1)	33 591 (83.5)*	47 331 (79.2)*
Black	461 (14.4)	129 (11.6)	17 (9.0)	4591 (11.4)	7128 (11.9)
Others	165 (5.2)	41 (3.7)	13 (6.9)	2034 (5.1)	5313 (8.9)
Pathological differentiation, n (%)					
Well/moderately	51 (1.6)	41 (3.7)*	27 (14.3)*	17 609 (43.8)*	29 110 (48.7)*
Poorly/undifferentiated	3146 (98.4)	1070 (96.3)	162 (85.7)	22 607 (56.2)	30 662 (51.3)
Primary, n (%)					
Upper lobe	1861 (58.2)	664 (59.8)	92 (48.7)*	22 401 (55.7)*	35 398 (59.2)*
Middle lobe	139 (4.3)	51 (4.6)	10 (5.3)	1500 (3.7)	2897 (4.8)
Lower lobe	761 (23.8)	268 (24.1)	71 (37.6)	11 670 (29.0)	16 246 (27.2)
Others	436 (13.6)	128 (11.5)	16 (8.5)	4645 (11.6)	5231 (8.8)
T, n (%)					
T1	602 (18.8)	292 (26.3)*	53 (28.0)*	7693 (19.1)	17 149 (28.7)*
T2	919 (28.7)	302 (27.2)	61 (32.3)	11 337 (28.2)	16 933 (28.3)
T3	549 (17.2)	186 (16.7)	36 (19.0)	6936 (17.2)	9777 (16.4)
T4	1127 (35.3)	331 (29.8)	39 (20.6)	14 250 (35.4)	15 913 (26.6)
N, n (%)					
N0	1331 (41.6)	561 (50.5)*	121 (64.0)*	18 900 (47.0)*	28 216 (47.2)*
N1	317 (9.9)	132 (11.9)	26 (13.8)	4619 (11.5)	6231 (10.4)
N2	1182 (37.0)	317 (28.5)	36 (19.0)	13 253 (33.0)	19 259 (32.2)
N3	367 (11.5)	101 (9.1)	6 (3.2)	3444 (8.6)	6066 (10.1)
M, n (%)					
M0	1886 (59.0)	726 (65.3)*	157 (83.1)*	29 134 (72.4)*	36 318 (60.8)
M1	1311 (41.0)	385 (34.7)	32 (16.9)	11 082 (27.6)	23 454 (39.2)
Stage, n (%)					
I	525 (16.4)	295 (26.6)*	78 (41.3)*	9053 (22.5)*	16 375 (27.4)*
II	320 (10.0)	154 (13.9)	35 (18.5)	5503 (13.7)	6143 (10.3)
III	1041 (32.6)	277 (24.9)	44 (23.3)	14 578 (36.2)	13 800 (23.1)
IV	1311 (41.0)	385 (34.7)	32 (16.9)	11 082 (27.6)	23 454 (39.2)
Surgery, n (%)					
No	2131 (66.7)	530 (47.7)*	59 (31.2)*	25 533 (63.5)*	34 003 (56.9)*
Yes	1066 (33.3)	581 (52.3)	130 (68.8)	14 683 (36.5)	25 769 (43.1)
Chemotherapy, n (%)					
No	1805 (56.5)	515 (46.4)*	129 (68.3)*	23 194 (57.7)	33 114 (55.4)
Yes	1392 (43.5)	596 (53.6)	60 (31.7)	17 022 (42.3)	26 658 (44.6)
Radiotherapy, n (%)					
No	1834 (57.4)	731 (65.8)*	142 (75.1)*	22 730 (56.5)	38 121 (63.8)*
Yes	1363 (42.6)	380 (34.2)	47 (24.9)	17 486 (43.5)	21 651 (36.2)
Overall survival (months)†					
Median	8.0	15.0*	35.0*	13.0*	19.0*
95% Confidence interval	7.4–8.6	13.3–16.7	25.5–44.5	12.7–13.3	18.6–19.4

*P*‐values were all less than 0.05 in the comparison of five pathological types.

*P‐value <0.0125 (corrected α value = 0.05/4 = 0.0125) when compared with LCC using Chi‐squared tests except †Log‐rank tests.

ADE, adenocarcinoma; BSC, basaloid squamous cell carcinoma; LCC, large cell carcinoma; LCNEC, large cell neuroendocrine carcinoma; NSCLC, non‐small cell lung cancer; SCC, squamous cell carcinoma.

LCC over the age of 70 was significantly more than LCNEC but less than BSC and SCC. LCC and other NSCLCs were more likely to occur in males, except ADE. Pathological differentiation of ADE and SCC was generally superior to LCC and subtypes of LCC. For T stage patients, there was no significant difference between LCC and SCC (*P* = 0.945), and stage T3/4 was more than 50%, which was higher than other NSCLCs. Lymph node and distant metastasis were more common in LCC than others. Therefore, the proportion of stage III/IV in LCC, 73.6%, was the highest. Patients with LCC undergoing surgery were lower, but radiotherapy was higher.

### Analyses of patient characteristics based on AJCC stage in LCC and survival analyses

For LCC, there was no statistical difference in the composition of the AJCC stage in different races, but the situation was contrary for age, sex, grade, and primary site, as shown in Table 2. In all age cohorts, stage III/IV patients were all over 65%, and the highest proportion were 82.9% from 50 years or younger. After the Spearman rank correlation test, the correlation coefficient was −0.083 (*P* = 0.000). It was shown that there was a significant negative correlation between age and stage, but it was very weak. In different stages, more than 65% of patients were over 60 at the time of diagnosis. Not only were there more male patients than females, but the proportion of stage IV patients was also higher. LCC, which was higher in grade or located in other primary sites (including the main bronchus, overlapping lesion of lung and lung, NOS), tended to be a later stage.

**Table 2:** Comparison of AJCC stage in different groups of LCC

Variables	Stage I	Stage II	Stage III	Stage IV	Total	*P*‐value
Age, n (%)						0.000
≤50	27 (10.5)	17 (6.6)	93 (36.2)	120 (46.7)	257 (100)	
51–60	79 (12.0)	58 (8.8)	239 (36.4)	280 (42.7)	656 (100)	
61–70	167 (16.4)	105 (10.3)	315 (30.9)	431(42.3)	1018 (100)	
71–80	189 (20.8)	97 (10.7)	277 (30.5)	344 (37.9)	907 (100)	
>80	63 (17.5)	43 (12.0)	117 (32.6)	136 (37.9)	359 (100)	
Sex, n (%)†						0.000
Female	255 (19.5)	133 (10.2)	421 (32.2)	497 (38.1)	1306 (100)	
Male	270 (14.3)	187 (9.9)	620 (32.8)	814 (43.0)	1891 (100)	
Race, n (%)						0.083
White	448 (17.4)	253 (9.8)	824 (32.0)	1046 (40.7)	2571 (100)	
Black	51 (11.1)	45 (9.8)	167 (36.2)	198 (43.0)	461 (100)	
Others	26 (15.8)	22 (13.3)	50 (30.3)	67 (40.6)	165 (100)	
Pathological differentiation, n (%)†						0.000
Well/moderately	22 (43.1)	5 (9.8)	9 (17.6)	15 (29.4)	51 (100)	
Poorly/undifferentiated	503 (16.0)	315 (10.0)	1032 (32.8)	1296 (41.2)	3146 (100)	
Primary, n (%)						0.000
Upper lobe	338 (18.2)	192 (10.3)	614 (33.0)	717 (38.5)	1861 (100)	
Middle lobe	29 (20.9)	18 (12.9)	38 (27.3)	54 (38.8)	139 (100)	
Lower lobe	137 (18.0)	88 (11.6)	234 (30.7)	302 (39.7)	761 (100)	
Others	21 (4.8)	22 (5.0)	155 (35.6)	238 (54.6)	436 (100)	

*P*‐value for Kruskal‐Wallis tests except †Wilcoxon tests.

LCC, large cell carcinoma.

**Figure 1:**
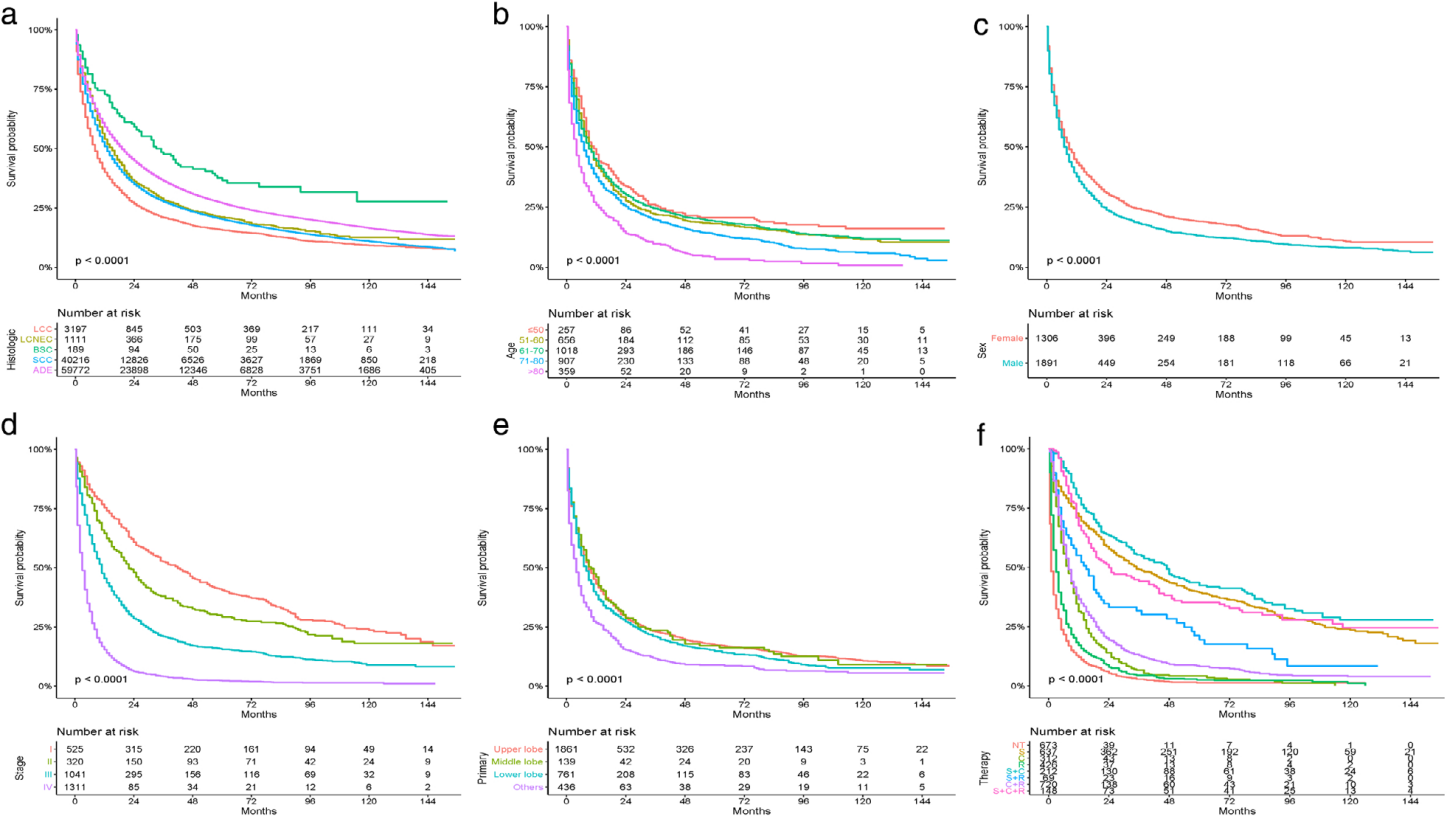
Kaplan‐Meier curves of overall survival between LCC and other NSCLCs. (**a**) (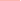) LCC, (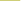) LCNEC, (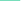) BSC, (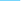) SCC, (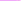) ADE, and for patients with LCC stratified by (**b**) age (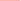) ≤50, (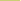) 51‐60, (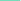) 61‐70, (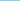) 71‐80, (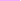) >80; (**c**) sex (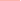) Female, (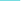) Male; (**d**) stage (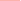) I, (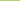) II, (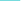) III, (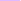) IV; (**e**) primary site (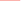) upper lobe, (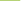) middle lobe, (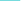) lower lobe, (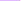) others; and (**f**) therapy (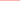) NT, (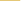) S, (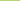) C, (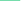) R, (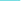) S+C, (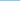) S+R, (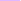) C+R, (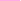) S+C+R. ADE, adenocarcinoma; BSC, basaloid squamous cell carcinoma; SCC, squamous cell carcinoma; LCC, large cell carcinoma; LCNEC, large cell neuroendocrine carcinoma; NSCLC, non‐small cell lung cancer; NT, no therapy; R, radiotherapy; S, surgery; C, chemotherapy.

Among 3197 LCC patients, 2800 patients had died, of which 2432 were tumor‐specific deaths. The median OS was eight months (95% confidence interval [CI]: 7.4–8.6). The one‐, three‐ and five‐year survival rate was 40%, 21%, and 15.6%, respectively. The median OS of stage I–IV patients were 42 months (95% CI: 34.4–49.6), 22 months (95% CI: 18.2–25.8), 11 months (95% CI: 9.8–12.2) and three months (95% CI: 2.7–3.3), respectively. OS was significantly worse in LCC (P<0.0001) than other pathological types (Fig. 1(a)). As shown in Fig. 1(f), patients who underwent surgery and comprehensive treatment including surgery survived better than others. The survival of patients with radiotherapy or chemotherapy alone was worse than chemotherapy combined with radiotherapy, which was worse than surgery combined with chemotherapy or radiotherapy.

### Analyses of LCC prognostic factors

Survival curves were constructed (Fig. 1 and Fig. S1) and log‐rank tests were performed to analyze possible prognostic factors in LCC. The OS was significantly worse in patients over 80 years. Male patients' prognosis was worse than females (*P* < 0.0001). Patients with well or moderately differentiated LCC tended to have later death time. Tumors located in the upper, middle, and lower lobe were superior to main bronchus or overlapping lesions in survival. T, N, and M stages were associated with prognosis. The later the stage, the worse the survival curves became, except overlapping survival curves of N2 and N3 (*P* = 0.143). There was no statistical difference among different races (*P* = 0.28) in OS. Surgery‐based comprehensive treatment was better than other treatments.

To analyze the impact of various factors on survival, we then constructed a Cox regression model. As shown in Table 3, in the univariate analysis, aging, males, poorly or undifferentiated, others in primary and later TNM stages were not conducive to survival. Any therapy and excision of lymph nodes were favorable factors in survival. Factors with *P* < 0.05 continued to be included in the multivariate analysis.

**Table 3:** Univariate and multivariate Cox proportional hazard analysis

	Univariate analysis		Multivariate analysis	
Variables	HR (95%CI)	*P*‐value	HR (95%CI)	*P*‐value
Age, n (%)				
≤ 50	Reference		Reference	
51–60	1.143 (0.975, 1.340)	0.0997	1.053 (0.897, 1.237)	0.525 250
61–70	1.131 (0.973, 1.316)	0.1097	1.143 (0.980, 1.332)	0.08785
71–80	1.384(1.189, 1.611)	2.71E‐05	1.373(1.175, 1.604)	6.76E‐05
>80	2.011 (1.693, 2.388)	1.77E‐15	1.635 (1.365, 1.958)	9.12E‐08
Race, n (%)				
White	Reference			
Black	1.022 (0.918, 1.137)	0.692		
Others	0.876 (0.739, 1.039)	0.128		
Sex, n (%)				
Female	Reference		Reference	
Male	1.181 (1.095, 1.274)	1.68E‐05	1.127 (1.044, 1.217)	0.00225
Pathological differentiation, n (%)				
Well/moderately	Reference		Reference	
Poorly/undifferentiated	1.471 (1.080, 2.002)	0.0143	1.073 (0.786, 1.4467)	0.655 06
Primary, n (%)				
Upper lobe	Reference		Reference	
Middle lobe	1.000 (0.832, 1.204)	0.9961	0.996 (0,827, 1.199)	0.964 01
Lower lobe	1.100 (1.006, 1.203)	0.0373	1.053 (0.962, 1.154)	0.261 83
Others	1.549 (1.388, 1.729)	5.21E‐15	1.124 (1.006, 1.256)	0.03955
T, n (%)				
T1	Reference			
T2	1.398 (1.248, 1.567)	8.05E‐09		
T3	1.604 (1.413, 1.822)	3.33E‐13		
T4	1.996 (1.788, 2.228)	< 2E‐16		
N, n (%)				
N0	Reference			
N1	1.324 (1.159, 1.512)	3.62E‐05		
N2	2.070 (1.900, 2.256)	< 2E‐16		
N3	2.267 (2.005, 2.563)	< 2E‐16		
M, n (%)				
M0	Reference			
M1	3.126 (2.889, 3.382)	<2E‐16		
Stage, n (%)				
I	Reference		Reference	
II	1.264 (1.075, 1.486)	0.00453	1.283 (1.088, 1.513)	0.00298
III	2.003 (1.773, 2.262)	<2E‐16	1.824 (1.586, 2.097)	< 2E‐16
IV	4.768 (4.228, 5.378)	<2E‐16	3.478 (3.008, 4.021)	< 2E‐16
Lymph nodes removed, n (%)				
No	Reference		Reference	
Yes	0.299 (0.274, 0.327)	<2E‐16	0.669 (0.569, 0.786)	1.06E‐06
Therapy, n (%)				
No therapy	Reference		Reference	
Only surgery	0.157 (0.139, 0.178)	<2E‐16	0.399 (0.327, 0.487)	< 2E‐16
Only chemotherapy	0.476 (0.416, 0.546)	<2E‐16	0.423 (0.368, 0.487)	< 2E‐16
Only radiotherapy	0.702 (0.621, 0.793)	1.55E‐08	0.691 (0.611, 0.782)	4.17E‐09
Surgery and chemotherapy	0.129 (0.107, 0.156)	<2E‐16	0.282 (0.222, 0.359)	< 2E‐16
Surgery and radiotherapy	0.262 (0.201, 0.343)	<2E‐16	0.493 (0.369, 0.660)	1.87E‐06
Chemotherapy and radiotherapy	0.389 (0.349, 0.434)	<2E‐16	0.408 (0.364, 0.457)	< 2E‐16
Trimodality therapy	0.160 (0.130, 0.197)	<2E‐16	0.281 (0.219, 0.360)	< 2E‐16

CI, confidence interval; HR, hazard ratio.

Considering the convenience of clinical application and that stage I–IV contained the information of T stage, N stage, and M stage, and that there was collinearity between stage IV and M1, stage I–IV was included in the multivariable analysis. Age, sex, grade, primary site, stage, therapy, and lymph nodes removed were included in the MVA (Table 3). Grade lost statistical significance (*P* = 0.655 06). Consistent with UVA, aging and later stage were unfavorable for survival. The risk of survival increased in patients 71–80 years and over 80 years, to which HR was 1.373 (95% CI, 1.175–1.604) and 1.635 (95% CI, 1.365–1.958), respectively. Main bronchus or overlapping lesions were adverse factors for survival. Lymph node resection significantly improved patient survival. The prognoses of patients undergoing surgery combined chemotherapy and trimodality therapy were better than other treatments, but the HRs were very close when radiotherapy was added, from 0.282 to 0.281, indicating that the effect of radiotherapy may be very subtle.

**Figure 2:**
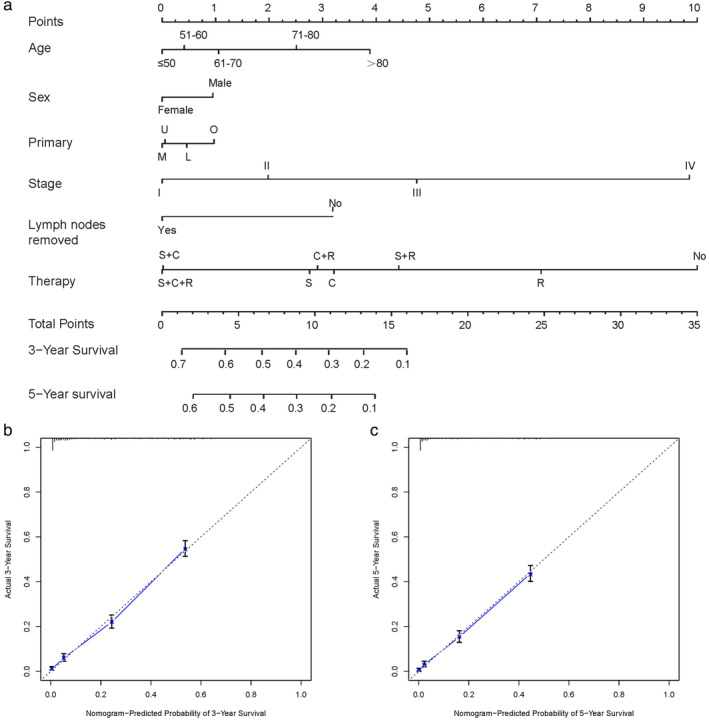
Nomogram and overall survival nomogram calibration curves. (**a**) Nomogram for prediction of three‐ and five‐year overall survival of patients with LCC. Calibration plots of the nomogram prediction of (**b**) Three‐year; and (**c**) Five‐year overall survival. The blue line represents the equality of the observed and predicted probability. C, chemotherapy; L, lower lobe; M, middle lobe; NT, no therapy; O, others; R, radiotherapy; S, surgery; U, upper lobe.

A nomogram was successfully constructed based on age, sex, primary site, stage, lymph nodes removed and therapy (Fig. 2). The C‐index of the nomogram was 0.754 (95% CI: 0.744–0.764). The slopes of calibration plots were all close to one, indicating that they were in good agreement with the actual three‐ and five‐year survival rates.

### Treatment of LCC in different stages

**Figure 3:**
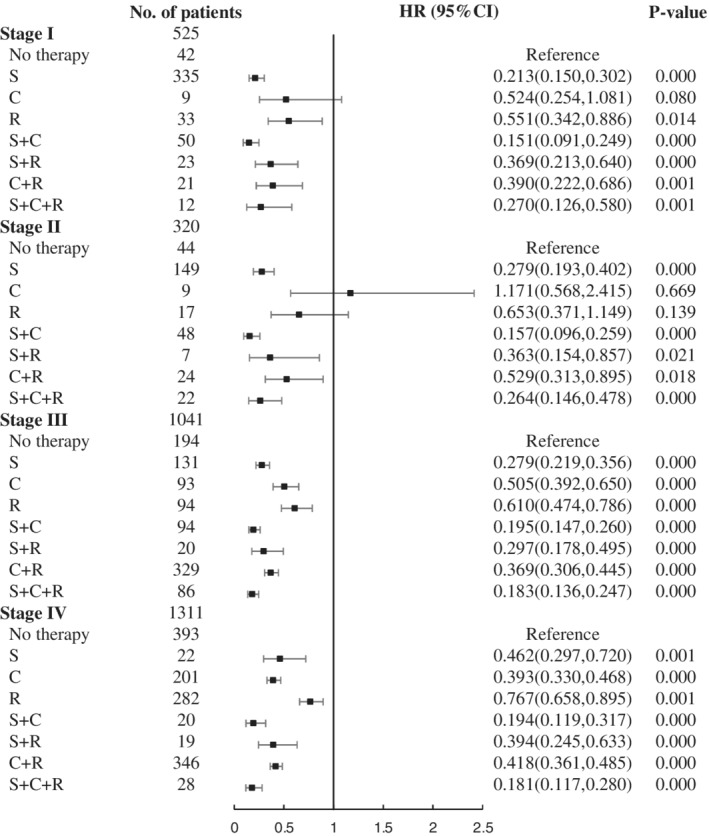
Forest plot of HRs for LCC in stage I–IV. C, chemotherapy; CI, confidence interval; HR, hazard ratio; LCC, large cell carcinoma; R, radiotherapy; S, surgery.

When confronted with LCC, what we can do is to choose the appropriate treatment. To further investigate the optimal treatment in different stages, analyses were performed on stage I–IV patients and the results are shown in Fig. 3. In patients with stage I/II, chemotherapy alone was not significantly different from no therapy (I: *P* = 0.080; II: *P* = 0.669), even increasing the mortality risk for stage II patients (HR: 1.171,95% CI: 0.568–2.415). However, surgery could contribute to around 75% reduction in risk of death compared with no therapy. The HR of the trimodality therapy was 0.183 (95% CI: 0.136–0.247) for stage III patients and 0.181 (95% CI: 0.117–0.280) for stage IV patients, showing distinct survival advantages. In different stages, surgery was beneficial to prognosis in varying degrees, even for stage IV patients (HR: 0.462,95% CI: 0.297–0.720). Surgery combined with chemotherapy in patients could further reduce the risk of death, in comparison with only surgery or chemotherapy alone in all stages. Surgery combined with radiotherapy in stage I–III patients not only did not expand the benefit compared with surgery, but may also cause damage to survival. However, for stage IV patients, radiotherapy added to surgery could probably be more beneficial, as indicated by the HR which changed from 0.462 (95% CI: 0.297–0.720) to 0.394 (95% CI: 0.245–0.633). Survival curves regarding the therapy of different stages are shown in Fig S2.

To further explore the role of surgery in stage IV patients, we evaluated the impact of surgery on survival (Fig. S3). The clinical characteristics of different therapy groups are shown in Table S1. There was no significant difference in age, sex, race, primary site, and differentiation in three groups. The survival curves showed that whatever therapy was combined with surgery could significantly improve survival, with *P*‐values of 0.0042, 0.0006, and 0.0071, respectively. In different surgical scenarios, when the surgical resection range is greater than one lobe, it may be helpful to patient prognosis. Sublobar treatment was not beneficial to survival, so far as to increase the risk.

## Discussion

LCC is one of the pathological types of NSCLC, lacking morphological or IHC evidence of adenocarcinoma or squamous carcinoma.^6^ Taking the criteria of WHO 2015 as standard, the diagnosis of LCC is limited to surgically‐resected tumors, and should not be applied to small biopsies or cytology. Subtypes of LCC are reclassified into different categories. LCNEC is in the group of neuroendocrine tumors, basaloid carcinoma in squamous cell carcinoma, and LELC in other and unclassified carcinomas. Clear cell carcinoma and rhabdoid phenotype are deemed as a cytological feature found in multiple histologic types. According to the IHC results, a part of LCC is now classified as adenocarcinoma or squamous cell carcinoma. Consequently, LCC has now become one of the rarest subtypes of NSCLC.^9^ Due to the low incidence and obvious changes in standard of classification, few studies have analyzed the clinical features and survival prognostic factors of LCC. The LCC patients included in this study did not meet the criteria of WHO 2015, as there was inadequate IHC information to eliminate LCC‐ADC and LCC‐SCC. Although the former subtypes were removed, there were still limitations in our study. Nevertheless, considering that not all surgical resection specimens of patients or IHC results could be obtained, and that former subtypes are excluded, our research is still of certain value for reference in clinical practice.

LCC and former subtypes of LCC, with partly similar clinical characteristics, have been reported to be more common in men and in the upper lobe. They were mainly poorly or undifferentiated, and different from SCC and ADE. The study of LCC that was rediagnosed according to the new criteria also revealed that the proportion of men and smokers was higher, and that it is more common in the periphery.^10^, ^11^, ^12^ The percentage of stage III/IV was highest in LCC, exceeding 70%. LCC patients were prone to distant metastasis and bone was the most frequent location in this study, followed by brain, lung and liver. Tonsillar^13^, ^14^ and intestinal^15^ metastases were rare but have also been reported in some case studies. Advanced stage LCC was more common in younger patients, in males, high grade, and in the main bronchus or overlapping lesions of the lung. The median OS of patients with LCC was reported to be eight months (95% CI: 7.4–8.6) and significantly worse than other NSCLCs. The research by Sun *et al*. in which patients received complete resection, found that the five‐year OS was not significantly different between classic LCC and LCNEC.^16^ Stage I patients, for which the median OS was 42 months (95% CI: 34.3–19.6), survived better than stage II–IV, implying that early detection and treatment is essential to prolong survival.

After log‐rank tests and Cox regression, we found that the elderly, male, later stage, and others in the primary site are risk factors for survival prognosis. Lymph node resection and any therapy could significantly improve prognosis. A study by Zhang *et al*. also showed that stage and different treatments were independent prognostic factors.^17^ In the nomogram established, the C‐index was 0.754, and the slope of the three‐year and five‐year survival rates of the calibration chart was close to one, which showed a good predictive effect.

Basic features are inherent, but treatment can be selected. Surgery‐based comprehensive treatment is an effective treatment for LCC, even in stage IV patients. Surgery combined with chemotherapy can further improve survival, but when combined with radiotherapy in stage I/II patients, it will increase the risk. In stage I/II patients, chemotherapy alone does not significantly improve survival, and the effect of only radiotherapy is also limited. In stage IV patients, either surgery or in combination with chemotherapy or radiotherapy, can dramatically improve survival. It may be beneficial if the resection range is greater than one lobe. However, due to the scarcity of surgical patients at stage IV, the results should be regarded warily, and further studies are necessary. For NSCLC with synchronized brain metastases, including LCC, patients have been reported to benefit from surgery of primary lung cancer and other treatments, with a one‐year survival rate of more than 60% and median OS of more than 20 months. LCC and lymph node metastasis are factors of poor prognosis.^18^, ^19^ For patients with synchronous isolated adrenal metastatic NSCLC, the median OS after lung surgery and complete adrenalectomy was 12 months, and the five‐year survival rate was around 20%.^20^, ^21^ Adrenal metastasis has more significant benefits than other oligometastasis for comprehensive treatment of combined surgery, without significant difference among various pathological types.^22^ Although the prognosis of patients with sync‐oligometastases is worse than oligo‐recurrence,^23^ surgery is still an option for a particular group.

After retesting specimens from patients previously diagnosed with LCC, 12%–42% met the new criteria.^5^, ^6^, ^11^, ^12^, ^24^, ^25^, ^26^ Under the new definition of LCC, little is known about the genetic changes. *KRAS* and *EGFR* mutations have been detected in the LCC‐null and LCC‐ADE. Inversely, there were no *EGFR* and *KRAS* mutations in LCC‐SCC, but there were low mutation rates in PIK3A, CDKN2A, and TP53.^5^, ^27^, ^28^ After reviewing several studies,^10^, ^11^, ^12^, ^25^, ^26^, ^27^, ^29^ the most common mutations in the LCC according to the new criteria were found to be *KRAS* and *EGFR* mutations. The *KRAS* mutation rate was about 11.6%, and the most common were G12C and G12V mutations. Generally speaking, *KRAS* mutation is a negative predictor of survival.^30^ Wang *et al*. showed that the five‐year survival rate of patients with *KRAS* mutations in LCC was lower than that of wild‐type (25.4% vs. 47.8%, *P* = 0.028).^11^ Studies on *KRAS* mutations have made progress. AMG 510,^31^ has been reported to be the first inhibitor of *KRAS G12C* mutation, and has worked well in NSCLC. In addition, the effect of AMG 510, when combined with chemotherapy or immunotherapy, is more prominent. We expect AMG 510 to play a role in LCC with the *KRAS* mutation. The *EGFR* mutation rate was around 6%, in which the rate of L858R mutation was highest. According to NCCN guidelines, targeted drugs are recommended for first‐line therapy for patients with *EGFR* mutation‐positive advanced or metastatic LCC.^32^

At the time of diagnosis, the majority of patients with LCC are elderly and at an advanced stage, which are all poor prognostic factors. According to our results, the LCC five‐year survival rate of LCC was only 15.6%. If the patients with positive outcomes of IHC were removed, the LCC five‐year survival rate would be lower. Steps can be taken to improve the survival of patients with LCC. On the one hand, LCC is common in smokers,^25^, ^33^ the elderly and males. Regular screening of high‐risk individuals could increase the early diagnosis rate. On the other hand, the best treatment should be chosen depending on the genetic changes, stage or status of patients. Also, a few clinical studies on immunotherapy included LCC.^34^ Although not explicitly analyzed, they still show that to some extent immunotherapy may affect LCC. Studies have shown that 44.4% of LCC patients expressed PDL1 in tumor cells,^11^ and PDL1 TPS ≥ 50% was found in 40% of LCC.^12^ According to NCCN guidelines, immunotherapy or immunotherapy combined with chemotherapy is recommended for first‐line therapy in patients with negative driver genes, with no contraindications for immunotherapy, and PD‐L1 expression ≥1%.^32^

The advantage of this study is that to the best of our knowledge it includes the greatest number of LCC patients. However, there are some limitations, and the results should be interpreted with caution. First, because of a lack of immunohistochemistry data, the LCC included in this study only excluded the subtypes of LCC removed by WHO 2015, but there was insufficient information for revaluation. Second, there was a lack of basic clinical information and specific medication information, such as smoking history, comorbidities, genetic testing, chemotherapy regimens, post‐treatment evaluation, targeted therapy or immunotherapy, etc. Third, the population included in the database and the processing of the data led to a selection bias.

In conclusion, we found that LCC was predominantly found in patients age over 60 years old, males, at later stages and with poor survival prognosis. Advanced stage LCC was more common in younger patients, males, high grade, and with primary cancer in the main bronchus, or overlapping lesions of the lung. Age, sex, stage, primary site, and therapy are closely related to survival. In stage I/II patients, compared with no therapy, surgery could reduce the risk of death by about 70%, and the survival benefit of chemotherapy or radiotherapy alone was not significant. In stage III/IV patients, a combination of surgery, chemotherapy, and radiotherapy showed considerable survival benefits. It is worth noting that in stage IV patients, survival could still be significantly improved if surgical resection involves more than one lobe.

## Disclosure

The author reports no conflicts of interest in this work.

## Supporting information


**Figure S1** Kaplan‐Meier curves of overall survival for patients with LCC stratified by (**a**) race; (**b**) grade; (**c**) T stage; (**d**) N stage; and (**e**) M stage.

**Figure S2** Kaplan‐Meier curves of overall survival of different therapy in LCC patients in (**a**) stage I; (**b**) stage II; (**c**) stage III; and (**d**) stage IV.

**Figure S3** Kaplan‐Meier curves of overall survival between surgery and nonsurgery in stage IV patients. (**a**/**d**) No therapy versus surgery or different surgical methods. (**b**/**e**) Chemotherapy versus chemotherapy combined with surgery or different surgical methods. (**c**/**f**) Radiotherapy versus radiotherapy combined with surgery or different surgical methods. (NT, no therapy; S, surgery; C, chemotherapy; R, radiotherapy; LL, excision or resection of less than one lobe; L/B: resection of one lobe or bilobectomy; P, pneumonectomy).

**Table S1** Comparison among different therapy in stage IV
