# Supplementary figures and images for: Clinical characteristics and prognosis of pulmonary large cell carcinoma: A population‐based retrospective study using SEER data
Source: Thorac Cancer. 2020 Apr 16;11(6):1522–32. doi: 10.1111/1759-7714.13420 (PMC7262949; doi:10.1111/1759-7714.13420)

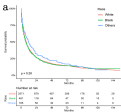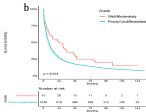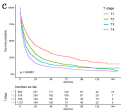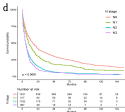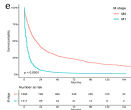

Supplement: Supplementary file 1 — Figure S1 Kaplan‐Meier curves of overall survival for patients with LCC stratified by (a) race; (b) grade; (c) T stage; (d) N stage; and (e) M stage. [file TCA-11-1522-s001.pdf]

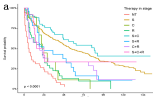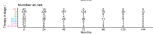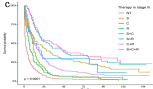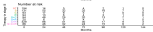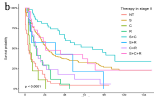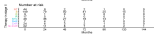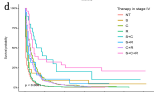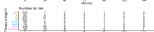

Supplement: Supplementary file 2 — Figure S2 Kaplan‐Meier curves of overall survival of different therapy in LCC patients in (a) stage I; (b) stage II; (c) stage III; and (d) stage IV. [file TCA-11-1522-s002.pdf]

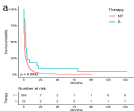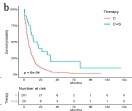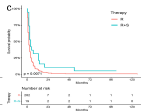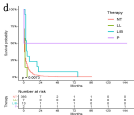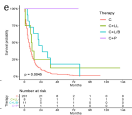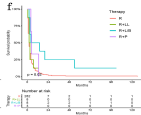

Supplement: Supplementary file 3 — Figure S3 Kaplan‐Meier curves of overall survival between surgery and nonsurgery in stage IV patients. (a/d) No therapy versus surgery or different surgical methods. (b/e) Chemotherapy versus chemotherapy combined with surgery or different surgical methods. (c/f) Radiotherapy versus radiotherapy combined with surgery or different surgical methods. (NT, no therapy; S, surgery; C, chemotherapy; R, radiotherapy; LL, excision or resection of less than one lobe; L/B: resection of one lobe or bilobectomy; P, pneumonectomy). [file TCA-11-1522-s003.pdf]
