# Supplementary material for: Clinical characteristics and prognosis of pulmonary large cell carcinoma: A population‐based retrospective study using SEER data
Source: Thorac Cancer. 2020 Apr 16;11(6):1522–32. doi: 10.1111/1759-7714.13420 (PMC7262949; doi:10.1111/1759-7714.13420)
Supplement: Supplementary file 4 — Table S1 Comparison among different therapy in stage IV [file TCA-11-1522-s004.pdf]

Supplement table Comparison among different therapy in stage IV

Variables	NT and S			C and S+C			R and S+R		
	NT (N=393)	S (N=22)	P-value	C (N=201)	C+S (N=20)	P-value	R (N=282)	R+S (N=19)	P-value
**Age,n(%)**									
≤50	24(6.1)	2(9.1)	0.397	16(8.0)	4(20.0)	0.340	19(6.7)	1(5.3)	0.154
51-60	60(15.3)	2(9.1)		46(22.9)	5(25.0)		66(23.4)	6(31.6)	
61-70	105(26.7)	4(18.2)		76(37.8)	8(40.0)		91(32.3)	10(52.6)	
71-80	123(31.3)	11(50.5)		51(25.4)	3(15.0)		78(27.7)	2(10.5)	
＞80	81(20.6)	3(13.6)		12(6.0)	0(0)		28(9.9)	0(0)	
**Sex,n(%)**									
Female	140(35.6)	5(22.7)	0.257	85(42.3)	9(45.0)	0.815	99(35.1)	9(47.4)	0.281
Male	253(64.4)	17(77.3)		116(57.7)	11(55.0)		183(64.9)	10(52.6)	
**Race,n(%)**									
White	302(78.6)	18(81.8)	0.915	164(81.6)	18(90.0)	0.795	224(79.4)	18(94.7)	0.375
Black	71(18.1)	3(13.6)		27(13.4)	2(10.0)		37(13.1)	1(5.3)	
Others	20(5.1)	21(5.1)		10(5.0)	0(0)		21(7.4)	0(0)	
**Grade,n(%)**									
Well/Moderately	5(1.3)	0(0)	1.000	0(0)	0(0)	1.000	3(1.1)	0(0)	1.000
Poorly/Undifferetiate	388(98.7)	33(100)		201(100)	20(100)		279(98.9)	19(100)	
**Primary,n(%)**									
Upper lobe	196(49.9)	11(50.0)	0.640	111(55.2)	12(60.0)	0.598	146(51.8)	14(73.7)	0.071
Middle lobe	19(4.8)	1(4.5)		7(3.5)	0(0)		15(5.3)	1(5.3)	
Lower lobe	89(22.6)	7(31.8)		53(26.4)	7(35.1)		62(22.0)	4(21.1)	
Others	89(22.6)	3(13.6)		30(14.9)	1(5.0)		59(20.9)	0(0)	

Notes: P-value for Chi-square tests or Fisher's exact tests.

Abbreviations: NT:no therapy; S:surgery; C:chemotherapy; R,radiotherapy
